# Draft genome sequence of *Streptomyces* sp. CC302I with non-canonical biosynthetic gene clusters for codon-readthrough activity

**DOI:** 10.1128/mra.01109-24

**Published:** 2025-01-21

**Authors:** Luisa M. Trejo-Alarcon, Pablo Cruz-Morales, Cuauhtémoc Licona-Cassani

**Affiliations:** 1 Industrial Genomics Laboratory, FEMSA Biotechnolgy Center, School of Engineering and Sciences, Tecnológico de Monterrey, Monterrey, México; 2 Novo Nordisk Foundation Center for Biosustainability, Danmarks Tekniske Universitet, Kongens Lyngby, Denmark; 3 Integrative Biology Research Unit, The Institute for Obesity Research, Tecnológico de Monterrey, Monterrey, México; Wellesley College Department of Biological Sciences, Wellesley, Massachusetts, USA

**Keywords:** *Streptomyces*, draft genome, biosynthetic gene clusters (BGCs), codon-readthrough activity

## Abstract

*Streptomyces* sp. CC302I was isolated from a highly oligothrophic environment. High-throughput screening shows high codon-readthrough activity for the isolate with no canonical biosynthetic gene cluster responsible. Here, we report the whole-genome assembly of *Streptomyces* sp. CC302I and a preliminary identification of biosynthetic gene clusters encoded in the genome.

## ANNOUNCEMENT

The *Streptomyces* genus is a prolific source of microbial compounds with wide applications in health and biotechnology. *Streptomyces* sp. CC302I was isolated from oligotrophic soil at the Cuatro Ciénegas Basin, Chihuahuan dessert, Mexico (26°50′25.1″ N, 102°08′01.7″ W). We performed a high-throughput bioactivity screening focusing on codon-readthrough (CR) activity and observed that *Streptomyces* sp. CC302I exhibits a significant CR signal, suggesting potential ribosome-inhibiting activity similar to gentamicin, G418, and paromomycin (1). In response, we conducted genome sequencing, and genomic characterization focused on specialized metabolites. Initial analysis did not reveal canonical biosynthetic gene clusters (BGCs) responsible for CR bioactivity.

The soil sample collected from 10 to 15 cm depth was isolated using International *Streptomyces* Project liquid medium 2 (ISP-2), supplemented with nalidixic acid (30 mg/mL) and cycloheximide (100 mg/mL) to inhibit Gram-negative and fungi growth. Plates were incubated at 28°C for 7 days. The CR assay was performed using a previously described method with minor modifications (2). Briefly, the microbial culture supernatant was obtained using 1L: 10g strach, 0.2g MgSO4, 0.02g CaCl2, 1g KH2PO4, 1g K2HPO4, 1g (NH4)2SO4, 0.05g FeCl)3 (BS) medium (used for antibiotic production [3]), incubated at 30°C for 7 days and centrifuged at 14,000 × *g* for 15 minutes. Fifty microliters of supernatants was analyzed in 96-well plates containing YEpRG transformants, and positive signals were determined by fluorescence as indicated. Genomic DNA was extracted from ISP-2 cultures (1 × 10⁶ spores/mL) using liquid nitrogen lysis and phenol:chloroform:isoamyl alcohol (25 : 24 : 1, vol/vol) extraction (4). The genome was sequenced using the Illumina MiSeq platform with 2 × 300  bp paired-end reads. Libraries were prepared using TruSeq Nano DNA library kit (Illumina, San Diego, CA, USA) following the manufacturer’s instructions. Sequencing yielded 9,230,969 paired-end reads. FastQC v.0.74 (5, 6) assessed quality, and Trimmomatic (6, 7) removed adapters, low-quality bases, and reads shorter than 200 bp. Assembly was performed with Unicycler v.0.4.8 on the BV-BRC server v.3.93.3 (8, 9), yielding 7,912,083 bp with a G + C content of 72.65% and depth coverage of 179.5, in 118 contigs with an *N*_50_ of 130,546 bp. Taxonomic characterization was obtained using the TYGS server (10). *Streptomyces* sp. CC302I has 92.6% Digital DNA-DNA Hybridization (dDDH) (G + C content difference = 0.2%, *δ* = 0.128) with *Streptomyces vinaceusdrappus*. Genome annotation using the National Center for Biotechnology Information Prokaryotic Genome Annotation Pipeline (11) identified 7,042 genes, including 6,969 coding sequences, 18 rRNA genes (one complete 5S, 16S, and 23S, and two partial 23S), 66 tRNA genes, and 3 ncRNA genes. BGCs were identified using antiSMASH v.7.0 (12) and BIGFAM v.1.0.0 (13) to assess completeness. All software used default parameters unless specified.

**Fig 1:**
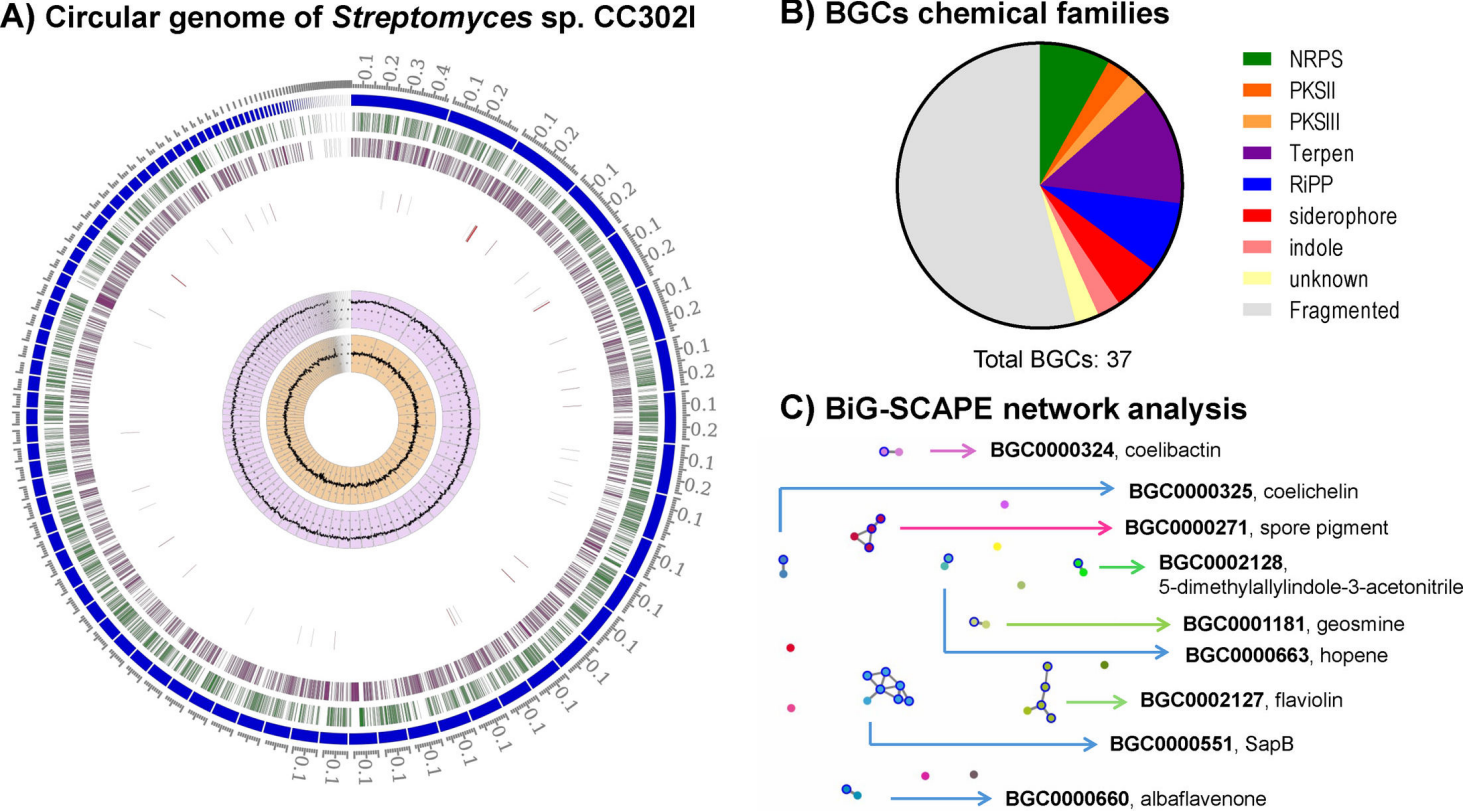
Graphical representation of the *Streptomyces* sp. CC302I genome. (**A**) Circular genome. From the center to the outside ring: Guanine-Cytosine (GC) skew (light pink), GC content (violet), antimicrobial resistance (AMR) genes (red), genes on the reverse strand (purple), genes on the forward strand (green), and contigs (blue). (**B**) Biosynthetic gene cluster chemical families (green, non-ribosomal peptide synthetase [NRPS]; orange, PKSII; light orange, PKSIII; purple, terpenes; blue, ribosomally synthesized and post-translationally modified peptides [RiPPs]; red, siderophore; light pink, indole; light yellow, unknown). The BGCs that are not completed are shown as fragmented in gray. (**C**) Biosynthetic Gene Similarity Clustering and Prospecting Engine (BiG-SCAPE) network analysis. The network connected nine BGCs that exhibited high similarity to known clusters in the MiBIG database, all coming from *Streptomyces coelicolor* and some that share similarity with other microorganisms as spore pigment (*Streptomyces avermitilis*, *Streptomyces collinus*, and *Streptomyces curacoi*), flaviolin (*Streptomyces clavuligerus* and *Saccharopolyspora erythraea*), and SapB (*Streptomyces griseus*, *Streptomyces albidoflavus*, *Streptomyces filamentosus*, *Saccharopolyspora erythraea*, and *Catenulispora acidiphila*). Nodes represent BGCs, while edges indicate similarity between clusters.

Figure 1 shows the genome, annotation, and BGC distribution. Seventeen complete BGCs (out of 37 annotated) were identified, covering non-ribosomal peptide synthetase (3), polyketide synthase types II (1) and III (1), terpene (5), ribosomally synthesized and post-translationally modified peptide (3), siderophore (2), indole (1), and unknown (1). Nine BGCs showed 80%–100% known metabolites in the MiBIG v.3.0 database (14), as marked in the figure. These BGCs may encode novel bioactivities with industrial and agricultural potential.

## Data Availability

The *Streptomyces* sp. CC302I genome sequence can be found at DDBJ/ENA/GenBank under accession number JBHOFE000000000. Raw reads are available at the National Center for Biotechnology Information Sequence Read Archive under accession number SRR30688880.

## References

[1] Dabrowski M, Bukowy-Bieryllo Z, Zietkiewicz E. 2018. Advances in therapeutic use of a drug-stimulated translational readthrough of premature termination codons. Mol Med 24. doi:10.1186/s10020-018-0024-7 PMC6016875 30134808

[2] Altamura E, Borgatti M, Finotti A, Gasparello J, Gambari R, Spinelli M, Castaldo R, Altamura N. 2016. Chemical-induced read-through at premature termination codons determined by a rapid dual-fluorescence system based on S. cerevisiae. PLoS One 11:e0154260. doi:10.1371/journal.pone.0154260 27119736 PMC4847774

[3] González-Salazar LA, Quezada M, Rodríguez-Orduña L, Ramos-Aboites H, Capon RJ, Souza-Saldívar V, Barona-Gomez F, Licona-Cassani C. 2023. Biosynthetic novelty index reveals the metabolic potential of rare actinobacteria isolated from highly oligotrophic sediments. Microb Genom 9:mgen000921. doi:10.1099/mgen.0.000921 36748531 PMC9973853

[4] Gallegos-Lopez S, Mejia-Ponce PM, Gonzalez-Salazar LA, Rodriguez-Orduña L, Souza-Saldivar V, Licona-Cassani C. 2020. Draft genome sequence of Streptomyces sp. strain C8S0, isolated from a highly oligotrophic sediment. Microbiol Resour Announc 9:01441–19. doi:10.1128/MRA.01441-19 PMC7118189 32241863

[5] Andrew S. 2010. FastQC: a quality control tool for high throughput sequence data. Available from: https://www.bioinformatics.babraham.ac.uk/projects/fastqc. Retrieved 19 Sep 2024.

[6] Giardine B, Riemer C, Hardison RC, Burhans R, Elnitski L, Shah P, Zhang Y, Blankenberg D, Albert I, Taylor J, Miller W, Kent WJ, Nekrutenko A. 2005. Galaxy: a platform for interactive large-scale genome analysis. Genome Res 15:1451–1455. doi:10.1101/gr.4086505 16169926 PMC1240089

[7] Bolger AM, Lohse M, Usadel B. 2014. Trimmomatic: a flexible trimmer for Illumina sequence data. Bioinformatics 30:2114–2120. doi:10.1093/bioinformatics/btu170 24695404 PMC4103590

[8] Wattam AR, Brettin T, Davis JJ, Gerdes S, Kenyon R, Machi D, Mao C, Olson R, Overbeek R, Pusch GD, Shukla MP, Stevens R, Vonstein V, Warren A, Xia F, Yoo H. 2018. Assembly, annotation, and comparative genomics in PATRIC, the all bacterial bioinformatics resource center. Methods Mol Biol 1704:79–101. doi:10.1007/978-1-4939-7463-4_4 29277864

[9] Wick RR, Judd LM, Gorrie CL, Holt KE. 2017. Unicycler: resolving bacterial genome assemblies from short and long sequencing reads. PLoS Comput Biol 13:e1005595. doi:10.1371/journal.pcbi.1005595 28594827 PMC5481147

[10] Meier-Kolthoff JP, Göker M. 2019. TYGS is an automated high-throughput platform for state-of-the-art genome-based taxonomy. Nat Commun 10:2182. doi:10.1038/s41467-019-10210-3 31097708 PMC6522516

[11] Tatusova T, DiCuccio M, Badretdin A, Chetvernin V, Nawrocki EP, Zaslavsky L, Lomsadze A, Pruitt KD, Borodovsky M, Ostell J. 2016. NCBI prokaryotic genome annotation pipeline. Nucleic Acids Res 44:6614–6624. doi:10.1093/nar/gkw569 27342282 PMC5001611

[12] Blin K, Shaw S, Augustijn HE, Reitz ZL, Biermann F, Alanjary M, Fetter A, Terlouw BR, Metcalf WW, Helfrich EJN, van Wezel GP, Medema MH, Weber T. 2023. antiSMASH 7.0: new and improved predictions for detection, regulation, chemical structures and visualisation. Nucleic Acids Res 51:W46–W50. doi:10.1093/nar/gkad344 37140036 PMC10320115

[13] Kautsar SA, Blin K, Shaw S, Weber T, Medema MH. 2021. BiG-FAM: the biosynthetic gene cluster families database. Nucleic Acids Res 49:D490–D497. doi:10.1093/nar/gkaa812 33010170 PMC7778980

[14] Terlouw BR, Blin K, Navarro-Muñoz JC, Avalon NE, Chevrette MG, Egbert S, Lee S, Meijer D, Recchia MJJ, Reitz ZL, et al.. 2023. MIBiG 3.0: a community-driven effort to annotate experimentally validated biosynthetic gene clusters. Nucleic Acids Res 51:D603–D610. doi:10.1093/nar/gkac1049 36399496 PMC9825592

